# Formation of Tankyrase Inhibitor-Induced Degradasomes Requires Proteasome Activity

**DOI:** 10.1371/journal.pone.0160507

**Published:** 2016-08-02

**Authors:** Nina Marie Pedersen, Tor Espen Thorvaldsen, Sebastian Wolfgang Schultz, Eva Maria Wenzel, Harald Stenmark

**Affiliations:** 1 Centre for Cancer Biomedicine, Faculty of Medicine, Oslo University Hospital, Oslo, Norway; 2 Department of Molecular Cell Biology, Institute for Cancer Research, Oslo University Hospital, Oslo, Norway; The University of Hong Kong, HONG KONG

## Abstract

In canonical Wnt signaling, the protein levels of the key signaling mediator β-catenin are under tight regulation by the multimeric destruction complex that mediates proteasomal degradation of β-catenin. In colorectal cancer, destruction complex activity is often compromised due to mutations in the multifunctional scaffolding protein Adenomatous Polyposis Coli (APC), leading to a stabilization of β-catenin. Recently, tankyrase inhibitors (TNKSi), a novel class of small molecule inhibitors, were shown to re-establish a functional destruction complex in APC-mutant cancer cell lines by stabilizing AXIN1/2, whose protein levels are usually kept low via poly(ADP-ribosyl)ation by the tankyrase enzymes (TNKS1/2). Surprisingly, we found that for the formation of the morphological correlates of destruction complexes, called degradasomes, functional proteasomes are required. In addition we found that AXIN2 is strongly upregulated after 6 h of TNKS inhibition. The proteasome inhibitor MG132 counteracted TNKSi-induced degradasome formation and AXIN2 stabilization, and this was accompanied by reduced transcription of *AXIN2*. Mechanistically we could implicate the transcription factor FoxM1 in this process, which was recently shown to be a transcriptional activator of *AXIN2*. We observed a substantial reduction in TNKSi-induced stabilization of AXIN2 after siRNA-mediated depletion of FoxM1 and found that proteasome inhibition reduced the active (phosphorylated) fraction of FoxM1. This can explain the decreased protein levels of AXIN2 after MG132 treatment. Our findings have implications for the design of *in vitro* studies on the destruction complex and for clinical applications of TNKSi.

## Introduction

The canonical Wnt signaling pathway is crucial for embryonic developmental processes and adult tissue homeostasis. Consequently, aberrations in this pathway were linked to human diseases and in particular cancer development [1]. The key mediator of the canonical Wnt signaling pathway is β-catenin, whose protein levels are under tight control by a multiprotein complex known as the destruction complex [2]. β-catenin is phosphorylated by this complex, which ultimately leads to its ubiquitin-proteasome-dependent degradation. In the presence of Wnt ligands the destruction complex becomes inactivated and β-catenin accumulates in the cytoplasm, translocates into the nucleus and initiates transcription of mitogenic target genes leading to cell proliferation. The core components of the destruction complex consist of Adenomatous Polyposis Coli (APC), axis inhibition protein 1 and 2 (AXIN1 and AXIN2) and the kinases glycogen synthase kinase 3 (GSK3) and casein kinase 1α (CK1α) [2, 3]. In the majority of colorectal cancers, APC is found to be mutated and the destruction complex thereby inactivated. Interestingly, overexpression of AXIN1 or AXIN2 can compensate for APC mutations and leads to the degradation of β-catenin in APC-mutant cell lines, such as SW480 colorectal cancer cells [4, 5]. AXIN has been shown to be the rate-limiting factor for destruction complex function in Xenopus egg extracts [6, 7] and its protein levels are tightly regulated by APC and by the poly-ADP-ribosyltransferases tankyrase 1 and 2 (TNKS1/2) [8, 9]. The tankyrase enzymes transfer ADP-ribose moieties onto AXIN1/2, marking it for degradation by the ubiquitin-proteasome system [10–12]. Inhibition of TNKS1/2 by small molecule inhibitors (TNKSi) has emerged as a promising new cancer therapeutic approach as it leads to stabilization of AXIN1/2 and a concomitant reduction in β-catenin protein levels and transcriptional activity *in vitro* and *in vivo* [8, 12–15]. Of note, *AXIN2* is also a target gene for β-catenin, adding another layer of AXIN2 regulation to the Wnt signaling pathway [16, 17].

In the current study, we sought to elucidate the consequences of combining TNKSi with proteasome inhibition, as proteasome inhibitors are extensively used in both clinical and research settings, often in combination with other inhibitors [18–20].

## Materials and Methods

### Antibodies, plasmids, and chemicals

The following reagents were used: rabbit anti-AXIN1 (C95H11), rabbit anti-AXIN2 (76G6) (Cell Signaling Technology), mouse anti-β-catenin (BD Transduction Laboratories); mouse anti-ubiquitin (Upstate / Millipore), mouse anti-active-β-catenin (05–665, Millipore); mouse anti-β-Actin (Sigma Aldrich), mouse anti-Calreticulin (Enzo lifesciences), mouse anti-Vinculin (HVIN-1, Sigma Aldrich), rabbit anti-FoxM1 (C-20, Santa Cruz), mouse anti-LaminA (Abcam), rabbit anti-p62 (MBL / Nordic Biosite). All secondary antibodies used for confocal microscopy studies were obtained from Jacksons ImmunoResearch Laboratories and secondary antibodies used for Western blotting were obtained from LI-COR Biosciences GmbH. Hoechst (Invitrogen). G007-LK (Gift from Stefan Krauss and Jo Waaler, Oslo, Norway); MG132 (Calbiochem); Dimethyl sulphoxide (DMSO), 3-Methyladenine (3-MA), Lactacystin, PhosSTOP (Sigma Aldrich); Epoximicin (Enzo lifesciences); Leupeptin (Peptanova Gmbh, Peptide Insitute, Japan). Quantitech mRNA primer pairs against TBP (QT00000721), AXIN2 (QT00037639) and FoxM1 (QT00000140) were obtained from Qiagen. FoxM1 siRNA (Sense: 5' GGACCACUUUCCCUACUUUUU-3', Antisense: 5' AAAGUAGGGAAAGUGGUCCUU 3' [21], and control siRNA (cat: D-001810-01), Dharmacon. siRNA transfections were performed using RNAiMax (Invitrogen) according to the manufacturer's protocol.

### Cell-based assays

SW480, COLO320, CaCo-2 and LS174T cell lines were purchased from ATCC. Upon receipt, cells were frozen, and individual aliquots were taken into cell culture, typically for analysis within 15 passages. Cells were grown in RPMI (SW480 and COLO320), DMEM (CaCo-2) or DMEM/F12 (LS174T) medium supplemented with 10% (SW480 and COLO320) or 15% (LS174T and CaCo-2) FBS and 1% penicillin/streptomycin. The stable SW480 cell line expressing GFP-TNKS1 was described earlier [22]. Testing for mycoplasma contamination was performed every sixth week. For inhibition of TNKS activity, cells were treated with 0.5 μM G007-LK for 6 h. DMSO was used as a control. For inhibition of proteasomal activity, cells were treated with 10 μM MG132, 25 nM Epoxomicin or 10 μM Lactacystin. Other inhibitors used were: 10 mM 3-Methyladenine (3-MA, autophagy inhibitor), or 300 μM Leupeptin (protease inhibitor) for indicated time points, either alone or in combination with G007-LK.

### Western blot analysis

Cells were rinsed in PBS and lysed in Laemmli lysis buffer (65.8 mmol/L Tris-HCl, pH 6.8, 2.1% SDS, 26.3% (w/v) glycerol, 0.01% bromophenol blue, dithiothreitol (DTT)). Equal amounts of whole cell lysate were separated by SDS-PAGE (Bio-Rad Laboratories) and blotted onto polyvinylidene difluoride membranes (Millipore). Immunodetection was performed with IRDye-conjugated secondary antibodies (LI-COR Biosciences). The Odyssey Imager system (LI-COR Biosciences) was used to scan all blots. Protein bands were quantified using the Odyssey software.

### Nucleo-cytoplasmic fractionation

Cells incubated with or without MG132 were washed with PSB and lysed in lysis buffer (0.1 M NaCl, 10 mM Na_2_HPO_4_, 1% Triton X-100, 1 mM EDTA (pH 7.4), 10 mM protease inhibitor cocktail, 2 mM NEM) and the lysates were left on ice for 20 min. The cell lysates were centrifuged at 14000 rpm for 10 min at 4°C to separate cytoplasmic (supernatant) and nuclear fraction (pellet). The fractions were then subjected for Western blotting.

### Confocal fluorescence microscopy

Cells grown on coverslips were permeabilized in PEM buffer (pH 6.8, 80 mM PIPES, 5 mM EGTA, 1 mM MgCl_2_x6H_2_O containing 0.05% saponin) for 5 min on ice before fixation in 3% paraformaldehyde for 15 min on ice and washed twice in PBS containing 0.05% saponin or permeabilized for 5 min with 0.5% Triton-X-100 in PBS, as indicated in the figure legends. The cells were then stained using the indicated primary antibodies for 1 h, washed three times in PBS/saponin, stained with secondary antibodies for 1 h, and washed three times in PBS. The coverslips were mounted in Mowiol containing 2 μg/ml Hoechst 33342 (Sigma-Aldrich). The cells were examined with a Zeiss LSM710 or LSM780 confocal microscope (Carl Zeiss MicroImaging GmbH) equipped with an Ar-Laser Multiline (458/488/514 nm), a DPSS-561 10 (561 nm) and a Laser diode 405-30CW (405 nm). The objective used was a Zeiss plan-Apochromat363/1.4 Oil DIC III. Image processing and visualization were performed with ZEN Software (Carl Zeiss MicroImaging GmbH), Photoshop CS4 (Adobe) and ImageJ (National Institutes of Health). All images were taken at fixed intensity settings below saturation.

### ScanR high-throughput microscopy

Cells were grown on coverslips and further processed for antibody staining as described for confocal microscopy samples. Images were automatically taken using the Olympus ScanR system with an UPLSAPO 40x/0.95 objective. All images were taken with the same settings and below pixel saturation. The Olympus ScanR analysis software was used to detect and count the number of GFP-TNKS1 puncta in samples treated with DMSO, G007-LK, MG132 or a combination of these, and to measure the nuclear intensity of β-catenin, FoxM1 and active β-catenin in DMSO versus MG132 treated samples.

### Quantitative real-time PCR of mRNA expression

mRNA expression analysis was done as described in [23]. Primers used in the study were AXIN2 (QT00037639), FoxM1 (QT00000140) and TBP (QT00000721).

### SDS immunoprecipitation

To investigate modifications of FoxM1 we did hot lysis immunoprecipitation as described in [24]. Shortly, cells were lysed in SDS (1%)–containing PBS, and immediately incubated at 100°C for 5 min, chilled on ice and homogenized using QIA-shredder column (QIAGEN). The lysates were added to protein G-coupled magnetic beads (Dynabeads, Life technologies / Thermo Fisher) loaded with rabbit anti-FoxM1 (C-20) antibody dissolved in 2x IP buffer (2% (vol/vol) Triton X-100, 0.5% (wt/vol) sodium deoxycholate, 1% (wt/vol) bovine serum albumin (BSA), 2 mM EDTA, 40 mM NaF, 2 mM NEM, 10 mM protease inhibitor cocktail (Sigma)). The beads and lysates were gently mixed for 1h at 4°C before the beads were washed in 1x IP buffer and eluted in 2x sample buffer, then subjected for Western blotting.

### Electron microscopy

SW480 were seeded on coverslips and treated with DMSO or MG132 for 6 hours before fixation in 2% glutaraldehyde in 0.1 M PHEM (240 mM PIPES, 100 mM HEPES, 8 mM MgCl_2_, 40 mM EGTA), pH 6.9, at room temperature for 40 min. Cells were post-fixed in osmium tetroxide, stained with tannic acid, dehydrated stepwise to 100% ethanol and flat-embedded in Epon. Serial sections (~100 nm) were cut on an Ultracut UCT ultramicrotome (Leica, Germany) and collected on formvar coated mesh-grids. Sections were observed at 80 kV in a JEOL-JEM 1230 electron microscope and images were recorded using iTEM software with a Morada camera (Olympus, Germany).

## Results and Discussion

### G007-LK-induced degradasome formation is counteracted by proteasome inhibition in SW480 cells

Inhibition of the TNKS enzymes by small-molecule inhibitors has previously been shown to reduce the aberrantly high levels of β-catenin in colorectal cancer cells such as SW480 cells by re-establishing a functional destruction complex [14, 15, 25]. Incubation of SW480 cells with the highly selective TNKSi G007-LK [26] for 6 h results in the formation of cytoplasmic puncta (degradasomes), which contain the destruction complex components AXIN1, AXIN2, APC, GSK3, βTrCP, TNKS1/2, β-catenin and phospho-β-catenin [22] and therefore most likely represent enlarged versions of the destruction complex, where β-catenin is phosphorylated and thereby earmarked for degradation in the proteasome. The formation of cytoplasmic puncta is most likely due to head-to-tail polymerization of AXIN molecules via their DIX domain [27, 28] and may also involve TNKS polymers [29–31].

**Fig 1:**
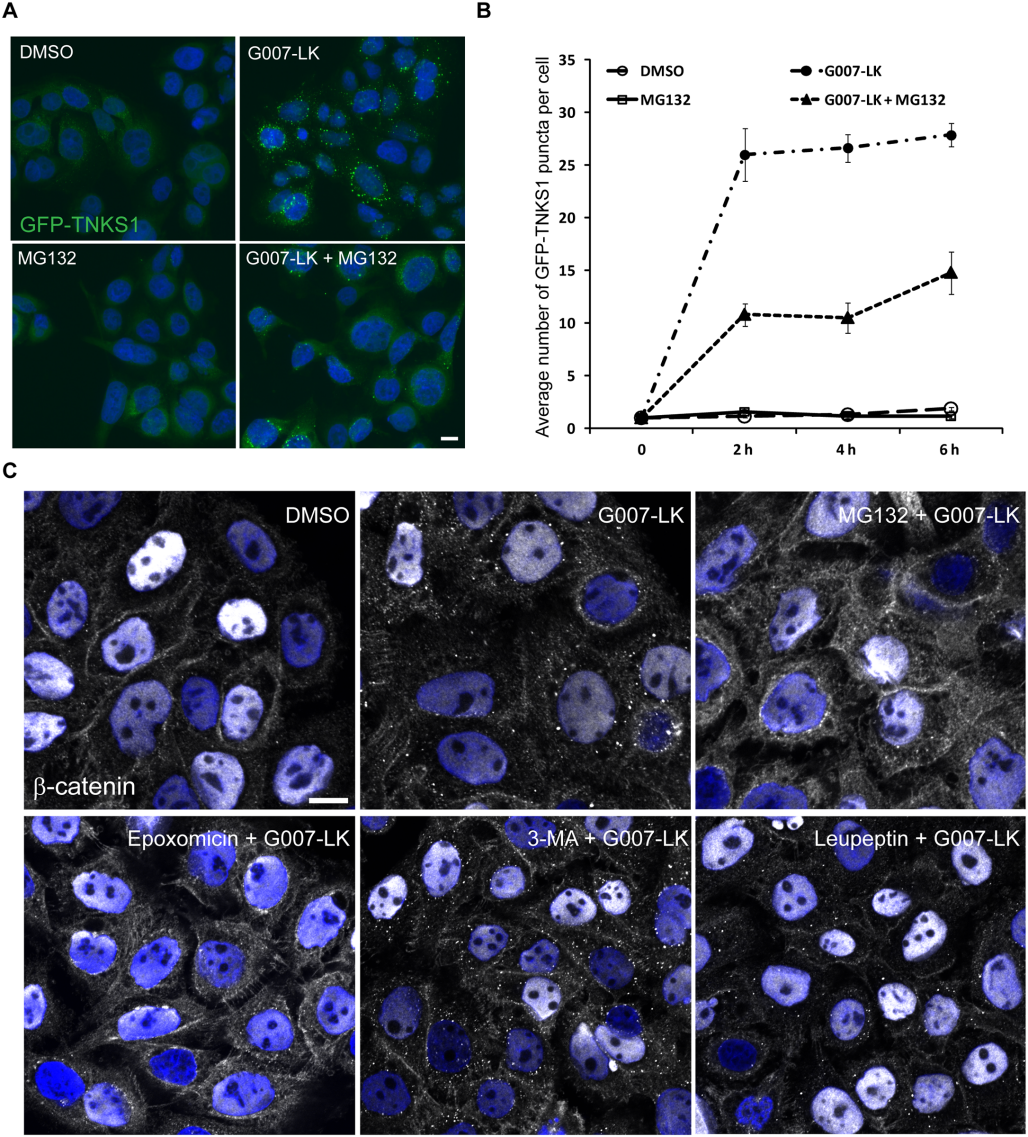
Inhibition of proteasome activity counteracts G007-LK induced degradasome formation. (A) SW480 cells stably expressing GFP-TNKS1 were treated with DMSO, G007-LK or MG132 alone or in combination for 6 h. Cells were PFA-fixed and imaged using an Olympus ScanR microscope. Representative images are shown. Hoechst in blue (nucleus). Scale bar: 10 μm. (B) Quantification of images in A. The average number of GFP-TNKS1 puncta per cell was analyzed and quantifications from 3 independent experiments, +/- SEM, are shown. ≥ 3000 cells were analyzed per condition in each experiment. There is a clear reduction in the number of GFP-TNKS1 puncta in cells treated with a combination of G007-LK and MG132 when compared to G007-LK alone. (C) SW480 cells were incubated with inhibitors of different degradation pathways: MG132 and Epoxomicin (proteasome inhibitors), 3-MA (autophagy inhibitor) and Leupeptin (lysosomal protease inhibitor), in combination with G007-LK for 6 h. Samples were PFA-fixed, saponin-permeabilized and prepared for confocal microscopy using an antibody against β-catenin (white). Hoechst in blue (nucleus). Note that only the proteasome inhibitors reduce the number of TNKSi-induced β-catenin puncta. Representative images are shown. Scale bar: 10 μm.

Surprisingly, the formation of degradasomes was reduced upon combination of G007-LK with the proteasome inhibitor MG132 for 6 h, as shown by high-throughput microscopy using an Olympus ScanR automated microscope (Fig 1A). The number of GFP-TNKS1 puncta was quantified using the ScanR analysis software and revealed a rapid induction of degadasomes after 2 h of incubation with G007-LK, while the combination of MG132 with TNKSi severely impaired degradasome formation (Fig 1B). To test whether this unexpected result could be reproduced with chemically unrelated proteasome inhibitors, we combined G007-LK with either 25 nM Epoxomicin (Fig 1C) or 10 μM Lactacystin (S1 Fig), respectively. Like with MG132, we observed a decrease in degradasome formation with these alternative proteasome inhibitors. On the other hand, lysosome inhibition (300 μM Leupeptin) or phosphatidylinositol-3 kinase class III inhibition (10 mM 3-methyladenine (3-MA)) did not interfere with degradasome formation (Fig 1C), indicating that specifically inhibiting the proteasome interferes with TNKSi-induced degradasome formation.

### TNKSi-induced AXIN2 stabilization is impaired upon proteasome inhibition

**Fig 2:**
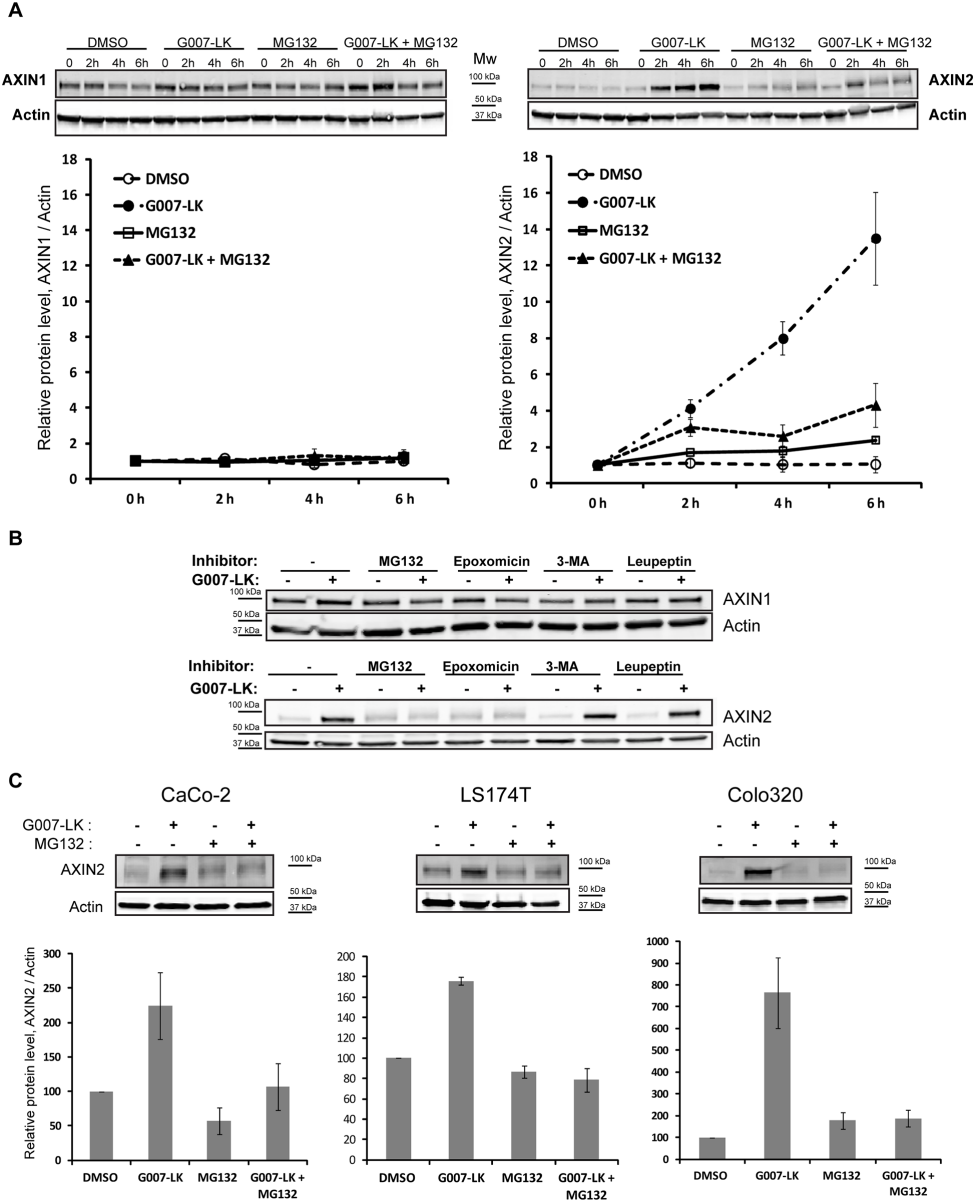
Proteasome activity is required for TNKSi-induced stabilization of AXIN2 protein level. (A) SW480 cells were incubated with DMSO, G007-LK or MG132, alone or in combination, for indicated time-points. Cells were then lysed and whole cell lysate was applied for Western blotting. Membranes were incubated with antibodies against AXIN1 and AXIN2. Actin was used as a loading control. One representative blot is shown. Quantifications of 3 independent experiments are shown below each blot, +/- SEM. Values at timepoint 0 h were normalized to 1 for each treatment and relative values of AXIN1/Actin or AXIN2/Actin are shown. (B) SW480 cells were incubated with the indicated inhibitors: MG132, Epoxomicin (proteasome inhibitors), 3-MA (autophagy inhibitor), Leupeptin (lysosomal protease inhibitor), alone or in combination with G007-LK for 6 h. Cells were then lysed and whole cell lysate was applied for Western blotting with antibodies against AXIN1 and AXIN2. Actin was used as a loading control. Again, the TNKSi-induced increase in AXIN2 protein levels was counteracted by the use of proteasome inhibitors (MG132, Epoxomicin) in combination with G007-LK, whereas AXIN1 protein levels were not significantly changed in any of the tested conditions. (C) CaCo-2, LS174T and Colo320 cells were incubated with DMSO, G007-LK, MG132 or a combination of G007-LK and MG132 for 6 h before cells were lysed and applied for Western blotting with an antibody against AXIN2. Actin was used as loading control. One representative blot is shown together with quantifications of three independent experiments, +/-SEM. Relative protein levels of AXIN2/Actin are shown.

TNKSi-induced stabilization of AXIN1 and/or AXIN2 is thought to mediate the re-establishment of functional destruction complexes [8]. Therefore, we investigated AXIN1 and AXIN2 protein levels during 6 h of G007-LK incubation with or without MG132. During this incubation time the protein levels of AXIN1 remained largely unaltered, while the AXIN2 levels strongly increased upon G007-LK treatment. This increase in AXIN2 was abrogated when G007-LK was combined with MG132 (Fig 2A), indicating that the TNKSi-induced stabilization of AXIN2 is impaired upon proteasome inhibition. The chemically unrelated proteasome inhibitor Epoxomicin prevented TNKSi-induced stabilization of AXIN2, but not AXIN1, similar to MG132, while 3-MA or Leupeptin did neither influence AXIN1 nor AXIN2 protein levels when co-incubated with G007-LK (Fig 2B).

Next, we tested whether this observation was restricted to SW480 cells or whether other colorectal cancer cell lines showed the same response on AXIN2 protein level. Indeed, CaCo-2, LS174T and Colo320 responded similarly to SW480 cells to the combination of G007-LK and MG132 with abolished AXIN2 stabilization, as shown by Western blotting and verified by quantifications (Fig 2C). We conclude that the lack of TNKSi-induced degradasome formation upon proteasome inhibition most likely depends on impaired stabilization of AXIN2.

### Proteasome activity inhibits transcription of *AXIN2*

**Fig 3:**
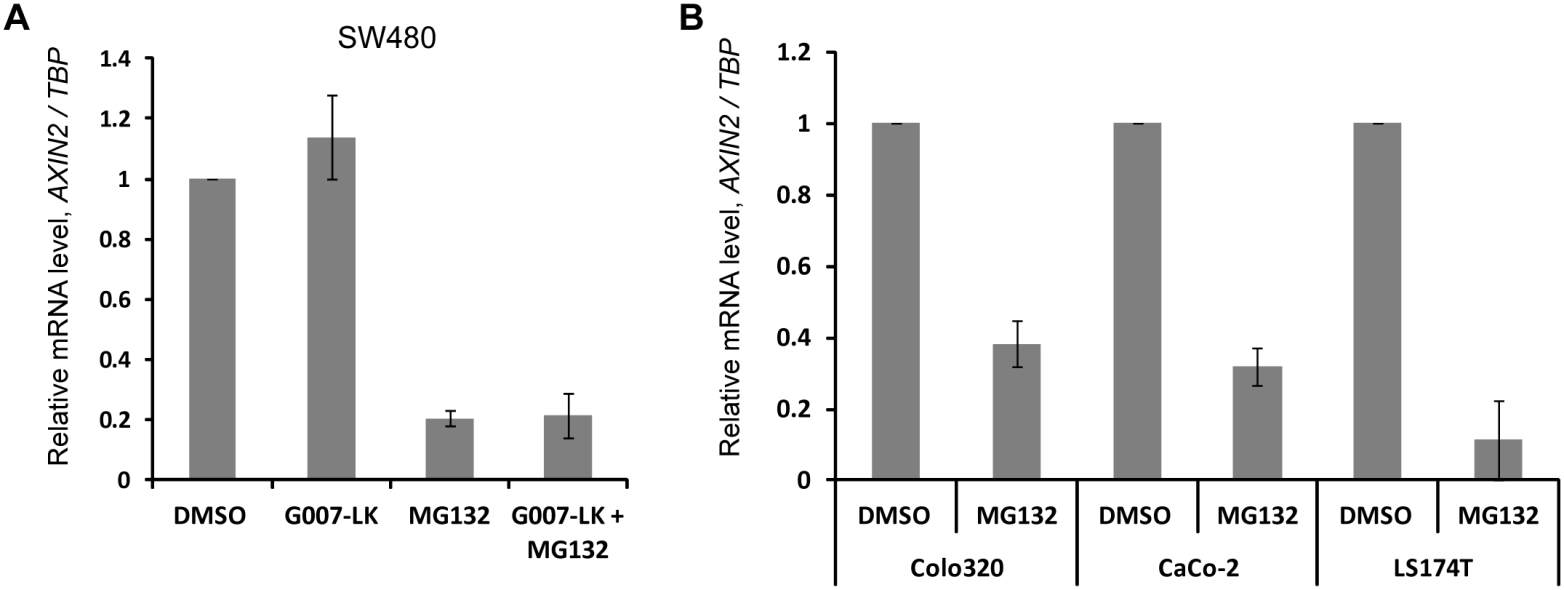
Proteasome activity is required for transcription of *AXIN2*. (A) SW480 cells were incubated with DMSO, G007-LK, MG132 or a combination of G007-LK and MG132 for 6 h before cells were washed and prepared for mRNA analysis as described in material and methods. Primer pairs for *AXIN2* and *TBP* (housekeeping gene) were used. Relative values of *AXIN2/TBP* mRNA levels are shown and values from the DMSO treated cells were normalized to 1. Three independent experiments are shown, +/- SEM. (B) Colo320, CaCo-2 and LS174T cells were incubated with DMSO or MG132 for 6 h and then prepared for analysis of *AXIN2* mRNA levels. *TBP* was used as a housekeeping gene. The graph shows relative *AXIN2/TBP* levels from three independent experiments, +/- SEM. For each cell line values from the DMSO treated sample were normalized to 1 for each cell line.

To distinguish whether the lack of AXIN2 protein stabilization upon combination of proteasome inhibition and G007-LK originates from altered mRNA levels or is due to a regulation on the protein level, we measured relative mRNA levels of *AXIN2* upon TNKS inhibition with or without MG132 by quantitative real-time PCR. MG132 led to a severe reduction in *AXIN2* mRNA levels in SW480 cells and this was not changed in the presence of both MG132 and G007-LK (Fig 3A). To verify whether MG132 had the same effect in other colorectal cancer cell lines with different APC and β-catenin mutation status, we performed similar experiments in LS174T, CaCo-2 and Colo320 cell lines. Indeed, we observed a reduction in *AXIN2* mRNA levels in all three cell lines upon MG132 incubation for 6 h (Fig 3B).

### Proteasome inhibition does not reduce nuclear β-catenin levels

**Fig 4:**
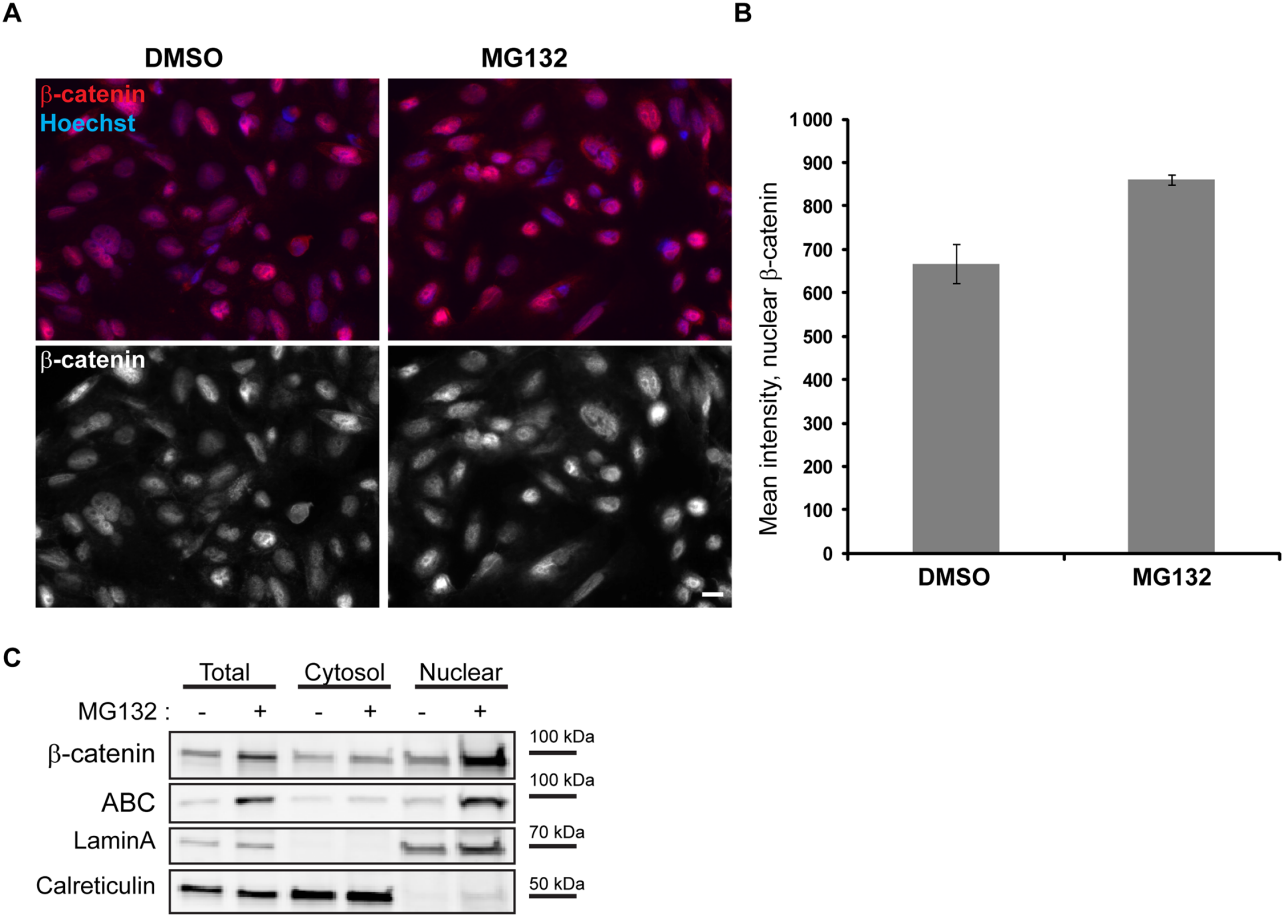
The nuclear localization of β-catenin is not reduced upon inhibition of proteasome activity. (A) SW480 cells were incubated with DMSO or MG132 for 6 h then fixed in PFA, permeabilized with Triton-X-100 and prepared for ScanR microscopy examination with an antibody against total β-catenin (white). Scale bar: 10 μm. (B) The graph shows quantification of β-catenin localization in SW480 cells incubated with DMSO or MG132 for 6 h. Quantifications are based on images taken with the Olympus ScanR high throughput microscope. 5x5 images were captured in two different areas per coverslip. Mean intensity of nuclear β-catenin per cell is show from three independent experiments. +/- SEM, and ≥ 10,000 cells were analyzed per condition. t test, p-value < 0.05. (C) Protein lysates of cells incubated with DMSO or MG132 for 6 h were fractionated into cytosolic and nuclear fractions and subjected for Western blotting with antibodies against β-catenin and active β-catenin (ABC). There is an increase in the total protein levels of β-catenin and active β-catenin (ABC) upon MG132 treatment and an accumulation of β-catenin and ABC in the nucleus. LaminA serves as a control for the nuclear fraction and Calreticulin for the cytosolic fraction.

As nuclear β-catenin promotes transcription of *AXIN2*, we investigated β-catenin localization in SW480 cells treated with either DMSO or MG132 by confocal microscopy and demonstrated a distinct staining of β-catenin in the nucleus under both conditions (Fig 4A). To investigate whether the amount of nuclear β-catenin in MG132-treated cells was reduced compared to control cells, we performed high-throughput microscopy that surprisingly revealed a slight increase in the mean fluorescence intensity of β-catenin in the nuclei of SW480 cells treated with MG132 (Fig 4B). To further interrogate whether the transcriptionally active fraction of β-catenin was altered upon MG132 treatment, we took advantage of an antibody specifically detecting non-phospho-β-catenin (i. e. active β-catenin, ABC). Quantitative fluorescence microscopy showed the same tendency for active as for total β-catenin (S2 Fig). Next, we undertook biochemical fractionation experiments to verify our imaging results. As expected, MG132 led to an accumulation of both β-catenin and active β-catenin in total protein lysates. Moreover, nuclear accumulation of both total and active β-catenin verified the results obtained by immunofluorescence (Fig 4C). Thus, changes in nuclear β-catenin levels cannot explain the decreased transcription of *AXIN2* mRNA upon proteasome inhibition.

### The proteasome-regulated transcription factor FoxM1 regulates *AXIN2* transcription

**Fig 5:**
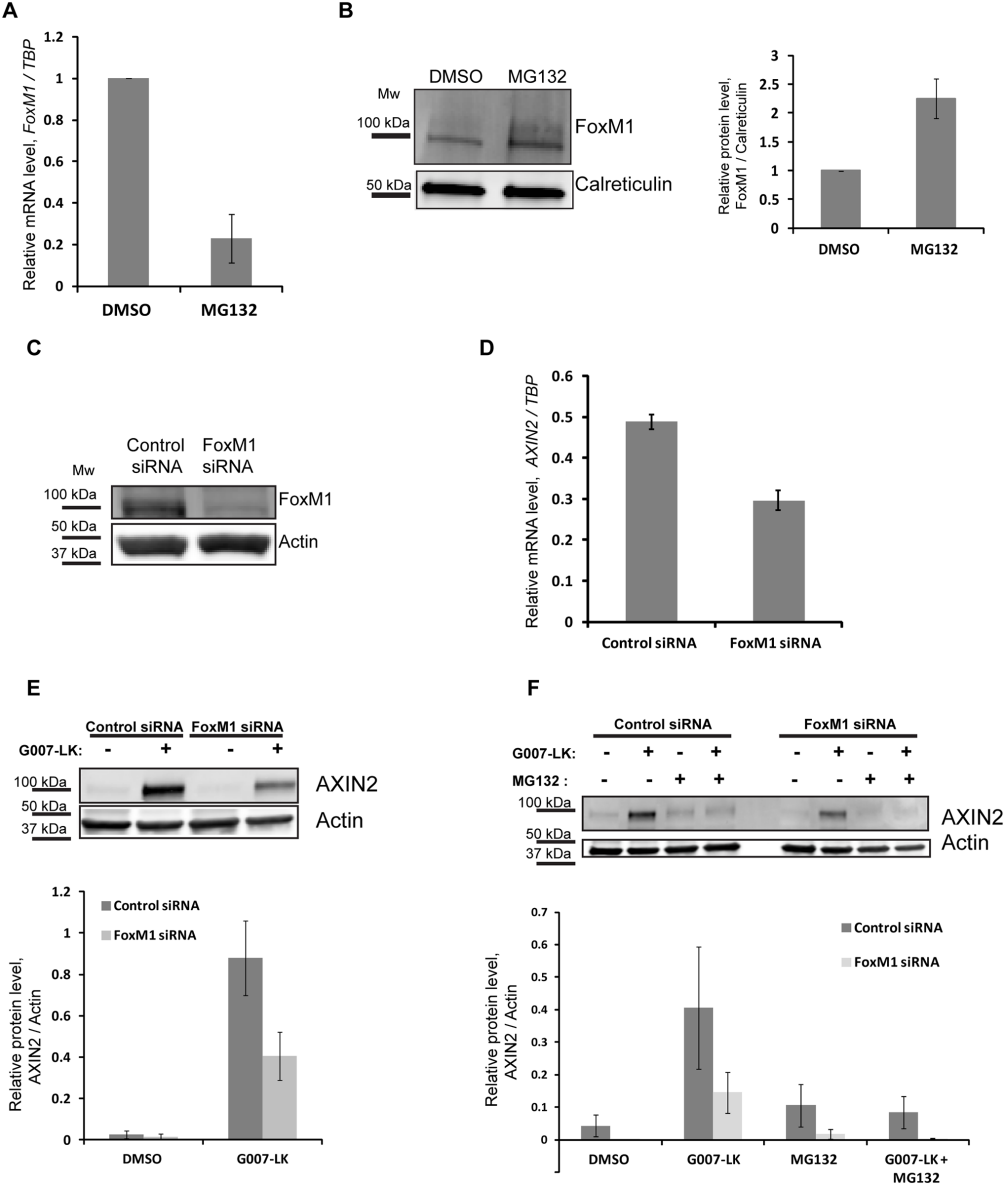
FoxM1 regulates the transcription of *AXIN2*. (A) SW480 cells were incubated with DMSO or MG132 for 6 h and then lysed and prepared for mRNA analysis with primers against *FoxM1* and *TBP* (housekeeping gene). Two independent experiments are shown +/- SD. (B) SW480 cells were incubated with DMSO and MG132 for 6 h. Cells were then lysed and whole cell lysate was applied for Western blotting. Membranes were incubated with an antibody against FoxM1. Calreticulin was used as a loading control. The graph shows quantification of three independent experiments +/- SEM, relative protein levels are shown, and the values in the DMSO sample were normalized to 1. (C) Western blot showing depletion of FoxM1 protein levels in SW480 cells after siRNA-mediated knock down (72 h). The membrane was incubated with an antibody against FoxM1 and Actin was used as a loading control. One representative blot is shown. (D) SW480 cells treated with FoxM1 or control siRNA (72 h) were lysed and prepared for analysis of *AXIN2* mRNA levels. *TBP* was used as a housekeeping gene. Three independent experiments are shown, +/- SEM. t test, p-value > 0.005. (E) SW480 cells treated with FoxM1 or control siRNA (72 h) were incubated with DMSO or G007-LK for the last 24 h and then lysed. Whole cell lysates were subjected to Western blotting and incubated with an antibody against AXIN2. Actin was used as a loading control. One representative blot is shown. The graph shows quantifications of three independent experiments, +/- SEM. (F) SW480 cells treated with FoxM1 or control siRNA (72 h) were incubated with DMSO, G007-LK, MG132 or a combination of G007-LK and MG132 for the last 6 h. Cells were lysed and whole cell lysates were subjected to Western blotting with an antibody against AXIN2. Actin was used as a loading control. One representative blot is shown. The graph shows quantifications of three independent experiments, +/- SEM.

Another regulator of *AXIN2* transcription is Forkhead box M1 (FoxM1). FoxM1 was previously shown to be a positive regulator of *AXIN2* mRNA levels by two different means: First, it can directly bind to and increase transcriptional activity of the *AXIN2* promotor region in developing lung epithelium [21]. Second, FoxM1 was reported to promote the nuclear localization of β-catenin and support β-catenin in binding to its target promotors, thereby indirectly controlling Wnt target-gene expression in glioma cells [32, 33]. Interestingly, FoxM1 was also shown to be negatively regulated by proteasome inhibition [34]. Proteasome inhibitors such as MG132, MG115 and bortezomib were shown to inhibit FoxM1 transcriptional activity and FoxM1 expression [34], and this seems to be mediated by stabilization of a negative regulator of FoxM1, namely HSP70 [35]. However, Chen and coworkers [32] report an increase in FoxM1 protein levels upon proteasome inhibition. Due to these conflicting reports, which most likely result from use of different cell lines and/or incubation protocols, we investigated FoxM1 mRNA and protein levels in SW480 cells with our experimental setup. We observed a substantial reduction of *FoxM1* mRNA levels upon 6 h MG132 treatment (Fig 5A). However, we detected a ~2.5 fold increase of FoxM1 at the protein level (Fig 5B). We hypothesize that despite a decrease in *FoxM1* mRNA transcription, the protein turnover of FoxM1 is severely impaired by the proteasome inhibition, which leads to an accumulation of FoxM1 protein.

To investigate whether FoxM1 functions as a positive regulator of Wnt-target gene expression in our model system, we treated SW480 cells with siRNA against FoxM1, which efficiently reduced FoxM1 protein levels (Fig 5C). We observed a significant reduction in *AXIN2* mRNA levels upon knockdown of FoxM1 (Fig 5D), consistent with the published role of FoxM1 as both direct and indirect transcriptional activator of Wnt target gene expression [21, 33].

In order to test the contribution of FoxM1 to the TNKSi-induced AXIN2 stabilization, we depleted SW480 cells for FoxM1 and incubated with G007-LK. We observed a substantial reduction in the TNKSi-induced AXIN2 protein levels in cells depleted for FoxM1 as shown by Western blotting (Fig 5E), and the same effect was true when G007-LK was combined with MG132 in FoxM1 depleted cells (Fig 5F). This confirms the role of FoxM1 in the regulation of AXIN2 protein levels.

### Posttranslational modifications of FoxM1 are altered upon proteasome inhibition

It was reported recently that USP5-mediated deubiquitination is required in order to promote translocation of FoxM1 into the nucleus, where it mediates interaction of β-catenin with target gene promotors [32]. We therefore hypothesized that proteasome inhibition may alter the nuclear translocation of FoxM1.

**Fig 6:**
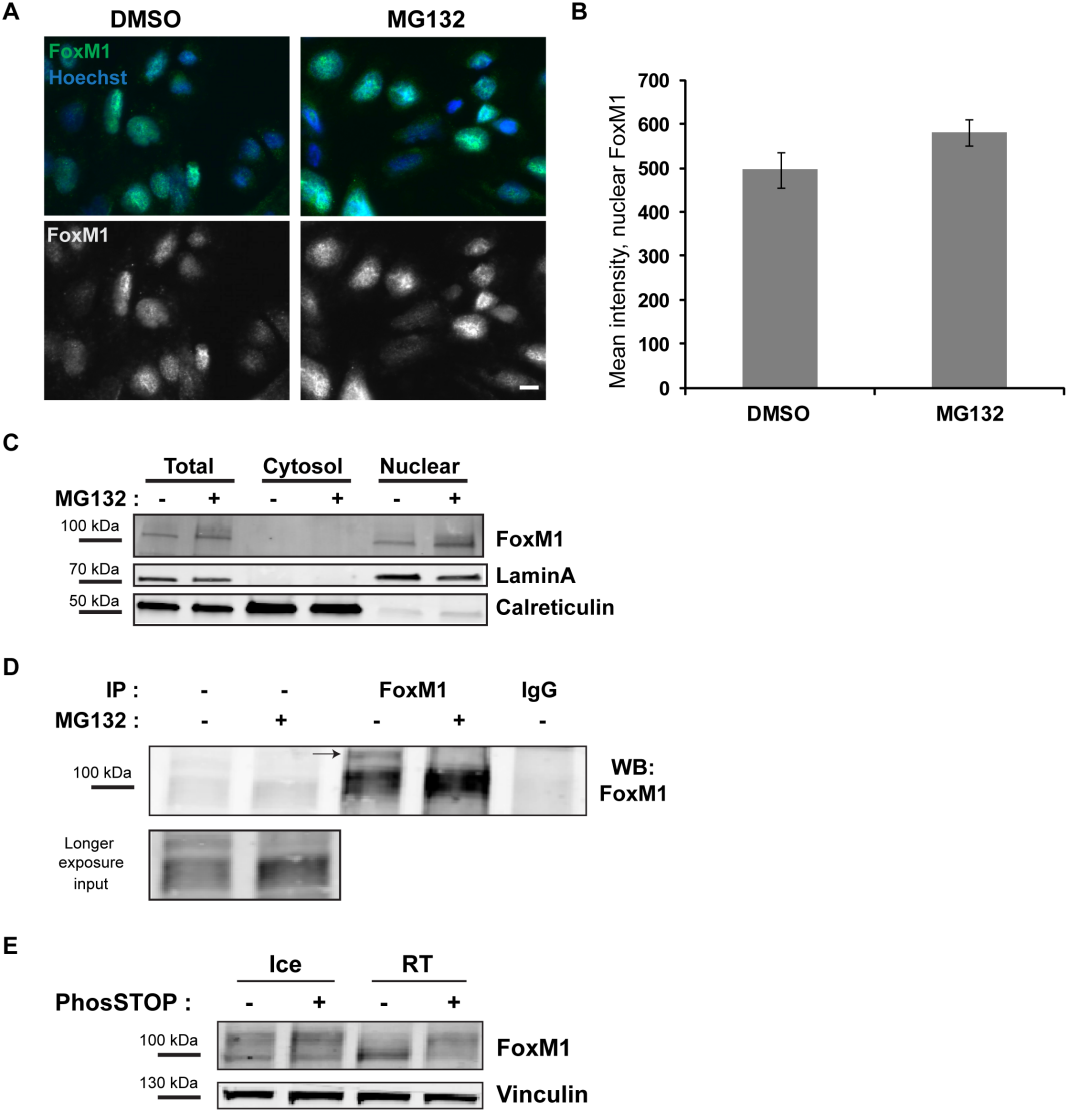
Proteasome activity alters the phosphorylation status of FoxM1. (A) SW480 cells were incubated with DMSO or MG132 for 6 h, pre-permeabilized with saponin on ice for 5 min, then fixed in PFA, Triton-X-100 permeabilized and prepared for ScanR microscopy examination with an antibody against FoxM1 (green and white). Scale bar: 10 μm. (B) The graph shows quantification of FoxM1 localization in SW480 cells incubated with DMSO or MG132 for 6 h. Quantifications are based on images taken with an Olympus ScanR high throughput microscope. 5x5 images were captured in two different areas per coverslip. Mean intensity of nuclear FoxM1 per cell is shown from three independent experiments, +/- SEM, ≥ 10,000 cells were analyzed per condition. t test, p-value > 0.05. (C) Protein lysates of cells incubated with DMSO or MG132 for 6 h were fractionated into cytosolic and nuclear fractions and separated on a 4–20% gradient gel. Western blotting with antibody against FoxM1 shows an increase in the total protein level of FoxM1 upon MG132 treatment and an accumulation of FoxM1 in the nucleus. LaminA serves as a control for the nuclear fraction and Calreticulin for the cytosolic fraction. (D) SDS immunoprecipitation of SW480 cells treated with DMSO (-) or MG132 (+) for 6 h was done as described in material and methods. Samples were separated on a 10% gel. A higher molecular weight band of FoxM1 probably representing posttranslational modifications of FoxM1 was detected in the DMSO treated sample (arrow) and disappeared upon MG132 treatment. Protein G beads coupled with rabbit anti-IgG was included as a control for unspecific binding. (E) SW480 protein lysates were incubated for one hour either on ice or at RT with or without PhosSTOP (phosphatase inhibitor mix). The higher molecular weight band, which was observed after running the protein lysates on a 10% gel for an extended period of time to allow proper size separation, disappeared in the absence of phosphatase inhibitors at room temperature. Vinculin was used as a loading control.

In order to investigate the subcellular localization of FoxM1, we performed immunofluorescence stainings for FoxM1 followed by high-throughput microscopy to quantify the nuclear fluorescence intensity of FoxM1. FoxM1 could be detected in the nuclei of SW480 cells after MG132 treatment (Fig 6A and 6B). Knockdown experiments verified the specificity of the FoxM1 antibody in immunofluorescence stainings (S3 Fig). To substantiate our findings we performed biochemical fractionation experiments, which showed the same tendency of a predominant localization of FoxM1 in the nucleus upon proteasome inhibition (Fig 6C). These data indicate that FoxM1 localization cannot explain the reduced transcription of *AXIN2* upon proteasome inhibition, irrespective of a possible ubiquitination of FoxM1.

Besides ubiquitination, FoxM1 is regulated by various phosphorylation events and all phosphorylation events were shown to stabilize and activate FoxM1 [36–40]. To investigate the phosphorylation status of FoxM1 in the absence (DMSO) or presence of proteasome inhibition (MG132), we performed a hot-lysis immunoprecipitation of FoxM1. Lysing cells in PBS containing 1% SDS with an immediate incubation at 100°C for 5 min leads to the instantaneous denaturation of all proteins (including phosphatases) and enables the investigation of posttranslational modifications of the immunoprecipitated proteins. We observed a higher molecular weight band in the DMSO-treated conditions, which was absent in the immunoprecipitate of the MG132 treated cells (Fig 6D). To investigate further whether this band could represent phosphorylated FoxM1, we incubated SW480 protein lysates with or without phosphatase inhibitor mix (PhosSTOP) for 1 h at room temperature. In the absence, but not in the presence, of the phosphatase inhibitor mix a high molecular weight band disappeared and the lower band increased in intensity, indicating that the higher molecular weight band is indeed phosphorylated FoxM1 (Fig 6E). We therefore suggest that despite FoxM1 localizing to the nucleus after MG132 treatment, it is dephosphorylated and thus transcriptionally less active upon proteasome inhibition. As most kinases phosphorylating FoxM1 are cell cycle kinases (CDK4/6, PLK1, CyclinA/CDK, Chk2) [36–40], we speculate that proteasome inhibition might lead to cell cycle arrest, synchronizing the cell population in a cell cycle phase where these kinases are not active.

Knockdown of FoxM1 has less effect on *AXIN2* transcription compared to MG132 treatment (Figs 5D and 3A). This could either mean that FoxM1 depletion was incomplete or that another still unknown factor influences *AXIN2* mRNA transcription. Indeed, 72 h after siRNA transfection, a minor fraction of FoxM1 protein seemed to be left (Fig 5C). In addition we noticed a redistribution of p62 and ubiquitin after MG132 treatment in immunofluorescence stainings (S4A Fig). This prompted us to investigate the general cell morphology after 6 h of treatment with MG132 at an ultrastructural level by electron microscopy. Surprisingly, we found that various cellular organelles were clustered around the nucleus resulting in an organelle-depleted cytoplasm in a subset of MG132-treated cells (S4B Fig). Such a drastic morphological change in a subpopulation of MG132-treated SW480 cells could possibly lead to an additional inhibitory effect on transcription and/or translation of proteins, e.g. AXIN2. However, transcription of house keeping genes was unaffected as judged by quantitative real-time experiments. Furthermore, protein levels were unaltered for AXIN1, arguing against a general full inhibition of gene transcription and protein translation upon proteasome inhibition.

Taken together, a reduction of FoxM1 activity in combination with changed cellular morphology may explain the lack of TNKSi-induced AXIN2 stabilization upon proteasome inhibition and thus the lack of degradasome formation when TNKSi are combined with proteasome inhibitors. While our manuscript was in preparation, another publication [29] reported that proteasome inhibition prevents degradasome formation and leads to decreased association of the PARsylated AXIN and TNKS proteins, but also to a perinuclear enrichment of AXIN. This is in agreement with our data, and our findings provide a further mechanistic explanation by showing that proteasome inhibition decreases the fraction of phosphorylated and thus active FoxM1 and that the cell morphology starts to change after 6 h of proteasome inhibition, which has implications for cell biological research utilizing proteasome inhibitors.

Given that proteasome inhibitors (including MG132) are used as potential therapeutic agents in colorectal cancer [41, 42], these findings are relevant regarding combination therapies with TNKSi. The fact that proteasome inhibition counteracts TNKSi-induced degradasomes argues against combining proteasome inhibitors and TNKSi in cancer therapy.

## Supporting Information

S1 Fig SW480 cells stably expressing GFP-TNKS1 (white) were incubated with G007-LK alone or in combination with the proteasome inhibitor Lactacystin for 6 h, then washed, fixed in PFA, permeabilized with saponin and prepared for confocal microscopy. Hoechst in blue (nucleus). Representative images are shown. Scale bar: 10 μm.
(PDF)

S2 Fig (A) SW480 cells were incubated with DMSO or MG132 for 6 h then fixed in PFA, permeabilized with Triton-X-100 and prepared for ScanR microscopy examination with an antibody against active β-catenin (ABC, red and white). Scale bar: 10 μm. (B) The graph shows quantification of nuclear localization of ABC in SW480 cells incubated with DMSO or MG132 for 6 h. Quantifications are based on images taken with an Olympus ScanR high throughput microscope. 5x5 images were captured in two different areas per coverslip. Mean intensity of nuclear ABC per cell is shown. Two independent experiments are shown, +/- SEM, and ≥ 10,000 cells were analyzed per condition. t test: p-value > 0.05.
(PDF)

S3 Fig SW480 cells treated with FoxM1 or control siRNA (72 h) were fixed in PFA and stained with an antibody against FoxM1 (C-20). Reduced nuclear staining of FoxM1 in FoxM1-depleted cells confirms the specificity of this FoxM1 antibody.
(PDF)

S4 Fig (A) SW480 cells were incubated with DMSO or MG132 for 6 h and fixed with PFA. Permeabilization was done with 0.5% Triton-X-100 in PBS. We observed a pronounced relocalization of ubiquitin and of the autophagy-adaptor protein p62 to the perinuclear region upon MG132 treatment. Hoechst in blue. Scale bar: 10 μm. (B) SW480 were seeded on coverslips and treated with DMSO or MG132 for 6 h before fixation and processing for electron microscopy. MG132 leads to a redistribution of organelles in a perinuclear area of a subset of MG132 treated cells. Scale bars: 5000 nm (overview) and 500 nm (inset).
(PDF)
