# Supplementary figures and images for: Formation of Tankyrase Inhibitor-Induced Degradasomes Requires Proteasome Activity
Source: PLoS One. 2016 Aug 2;11(8):e0160507. doi: 10.1371/journal.pone.0160507 (PMC4970726; doi:10.1371/journal.pone.0160507)

S1 Fig

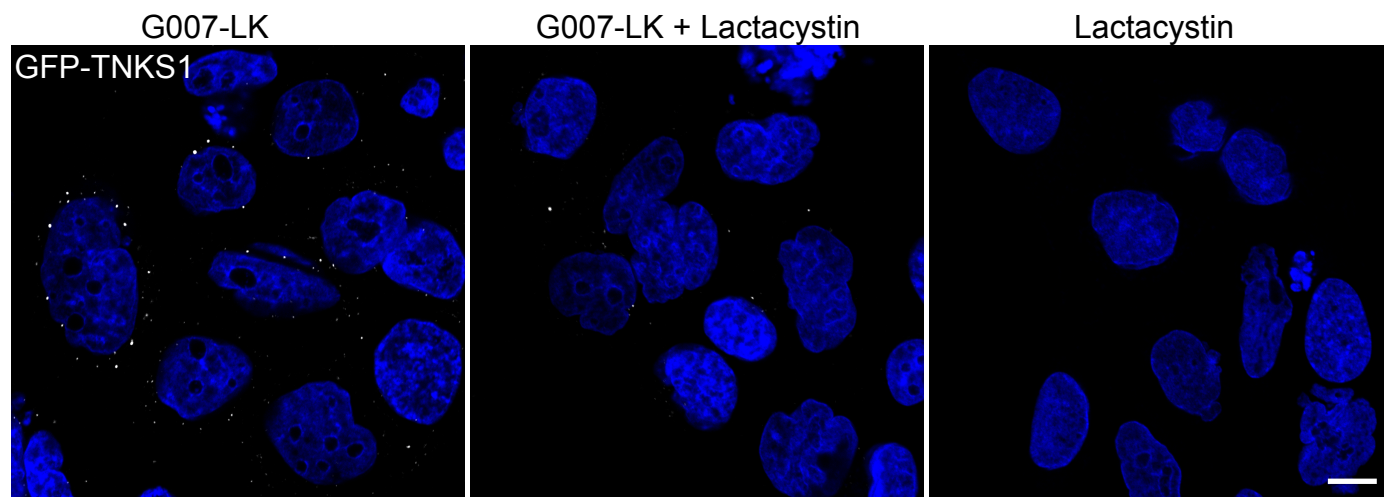

Supplement: S1 Fig — Hoechst in blue (nucleus). Representative images are shown. Scale bar: 10 μm. (PDF) [file pone.0160507.s001.pdf]

S2 Fig

A

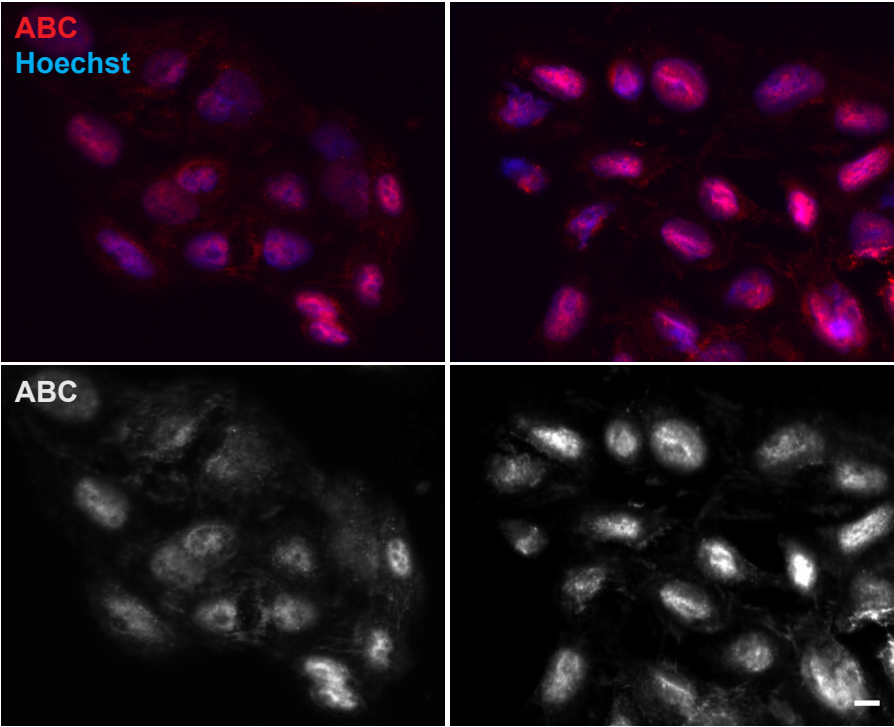

B

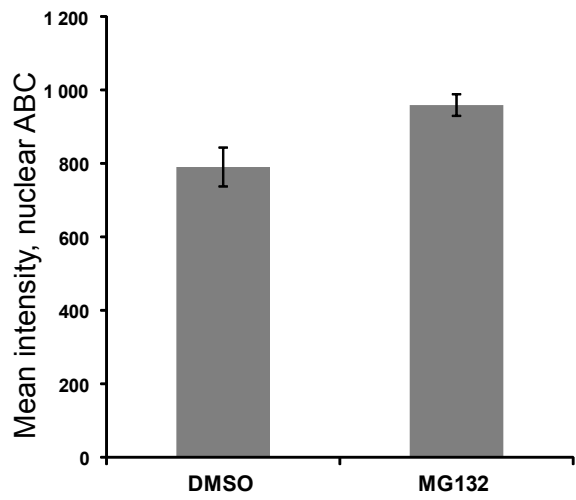

Supplement: S2 Fig — (A) SW480 cells were incubated with DMSO or MG132 for 6 h then fixed in PFA, permeabilized with Triton-X-100 and prepared for ScanR microscopy examination with an antibody against active β-catenin (ABC, red and white). Scale bar: 10 μm. (B) The graph shows quantification of nuclear localization of ABC in SW480 cells incubated with DMSO or MG132 for 6 h. Quantifications are based on images taken with an Olympus ScanR high throughput microscope. 5x5 images were captured in two different areas per coverslip. Mean intensity of nuclear ABC per cell is shown. Two independent experiments are shown, +/- SEM, and ≥ 10,000 cells were analyzed per condition. t test: p-value > 0.05. (PDF) [file pone.0160507.s002.pdf]

S3 Fig

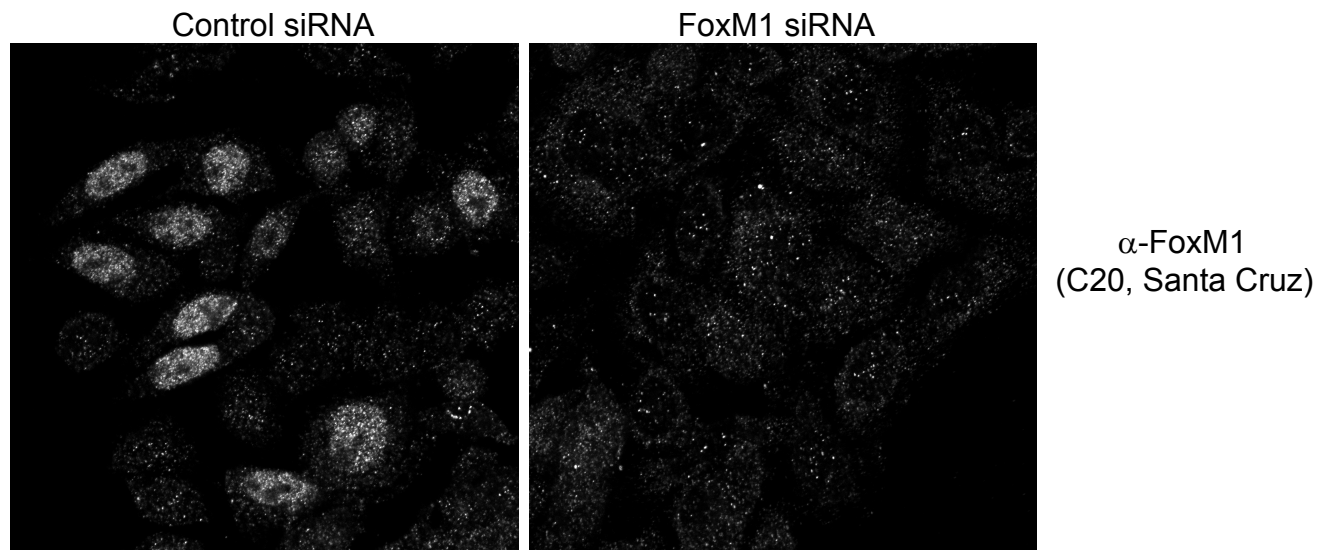

Supplement: S3 Fig — Reduced nuclear staining of FoxM1 in FoxM1-depleted cells confirms the specificity of this FoxM1 antibody. (PDF) [file pone.0160507.s003.pdf]

S4 Fig

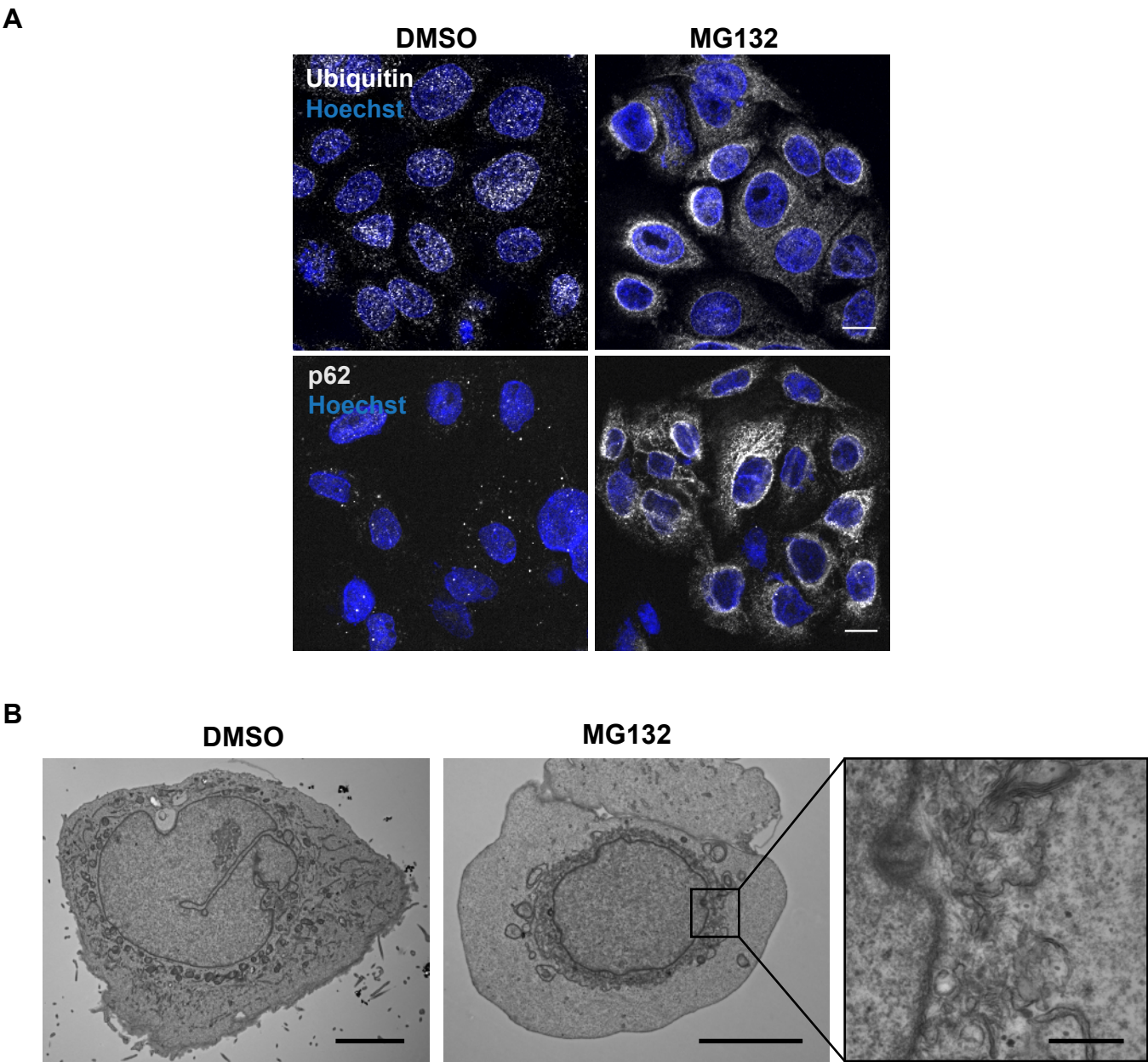

Supplement: S4 Fig — (A) SW480 cells were incubated with DMSO or MG132 for 6 h and fixed with PFA. Permeabilization was done with 0.5% Triton-X-100 in PBS. We observed a pronounced relocalization of ubiquitin and of the autophagy-adaptor protein p62 to the perinuclear region upon MG132 treatment. Hoechst in blue. Scale bar: 10 μm. (B) SW480 were seeded on coverslips and treated with DMSO or MG132 for 6 h before fixation and processing for electron microscopy. MG132 leads to a redistribution of organelles in a perinuclear area of a subset of MG132 treated cells. Scale bars: 5000 nm (overview) and 500 nm (inset). (PDF) [file pone.0160507.s004.pdf]
